# Glucocorticoids are double-edged sword in the treatment of COVID-19 and cancers

**DOI:** 10.7150/ijbs.58695

**Published:** 2021-04-10

**Authors:** Ruixin Yang, Yingyan Yu

**Affiliations:** Department of General Surgery of Ruijin Hospital, Shanghai Institute of Digestive Surgery, and Shanghai Key Laboratory for Gastric Neoplasms, Shanghai Jiao Tong University School of Medicine, 200025, Shanghai, China.

**Keywords:** COVID-19, Therapy, Glucocorticoids, Cancer

## Abstract

Glucocorticoids are important steroid hormones. As an outstanding scientific discovery, the scientist who discovered glucocorticoids was awarded the Nobel Prize in Physiology and Medicine in 1950. Cortisone (hydrocortisone) is a natural glucocorticoid, which is secreted with circadian rhythm by the cortical cells of adrenal glands. Physiologically, about 10-20 mg of hydrocortisone are secreted each day for maintaining homeostasis. Since the biological half-life of natural glucocorticoid is short, scientists developed various synthetic glucocorticoids including prednisone, prednisolone, methylprednisolone, triamcinolone, dexamethasone, betamethasone, and so on. These synthetic glucocorticoids are generated by modifying some structures based on the cortisone backbone, leading to extension of their biological half-life with stronger activities. In the face of severe infection, allergy, shock, trauma, pain, and other stresses, the demand for glucocorticoids increases dramatically. It is critical to supplement extra glucocorticoids to protect the biological functions of vital organs. However, the amount and duration of glucocorticoid administration need to be carefully adjusted, because a series of side effects may occur after long-term or high-dose usage of glucocorticoids. This review article will discuss the application of glucocorticoids in the treatment of patients with severe or critical COVID-19 and solid tumors of advanced stage. The controversy of using glucocorticoid in medical community will also be discussed. This review article will help doctors and basic researchers better understand the practical application of glucocorticoids.

## Classification of glucocorticoids

The discovery of glucocorticoid (cortisone) was awarded the Nobel Prize in Physiology and Medicine in 1950. Glucocorticoids are stress response hormones that bind to glucocorticoid receptor, and play critical roles in various physiological processes such as metabolic homeostasis, cognition, cell proliferation, reproduction, and inflammation. Glucocorticoid receptor (GR) belongs to the steroid-thyroid-retinoic acid nuclear receptor superfamily. In the absence of glucocorticoid binding, GR resides in the cytoplasm inactively binds to heat-shock proteins, FKBP51 and FKBP52. Upon glucocorticoid binding, GR undergoes conformational changes, dissociates from the chaperone proteins, and translocate into the nucleus, where it binds to promoter DNA of target genes, resulting in stimulation or suppression of the transcription of response genes. Natural glucocorticoids, namely cortisone or hydrocortisone, are cholesterol-derived hormones secreted by the adrenal glands and are named after their roles in maintaining glucose homeostasis. The release of glucocorticoids into blood circulation has systemic roles in immune response, metabolism, cell growth, development, and reproduction. Owing to their anti-inflammatory and immune-suppressive actions, glucocorticoids are among the most commonly prescribed drugs. Some autoimmune, inflammatory, and allergic disorders, including rheumatoid arthritis, lupus erythematosus, inflammatory bowel disease, transplant rejection and asthma are often treated with glucocorticoids 1, 2. Therefore, glucocorticoids are considered as a standard treatment in the above-mentioned diseases. Physiologically, an adult secretes 10-20 mg of glucocorticoids per day with circadian rhythms. The peak secretion is in the 6:00 to 8:00 a.m. The secretion of glucocorticoids are regulated by hypothalamic corticotropin, and secreted by adrenal cortex. In the face of stress, such as infections, trauma or surgery, the serum cortisol level could be increased to up to 150-200 mg. Since the cortisol was first synthesized in 1948, studies have been carried out with the aim of synthesizing steroids with a specific anti-inflammatory action 3. Pharmacological dose of glucocorticoids plays roles on anti-inflammation, anti-allergy, anti-shock, and immunosuppression. The medication of glucocorticoids should be given in accordance with the circadian rhythm in the early morning. Regarding the effect of anti-inflammation, glucocorticoids can quickly inhibit transcription of inflammatory cytokines, including IL-2, IL-3, IL-4, IL-5, IL-6, TNFα, GM-CSF, CCL1, CCL5, CCL11, and CXCL8 et al.^4^.

**Fig 1:**
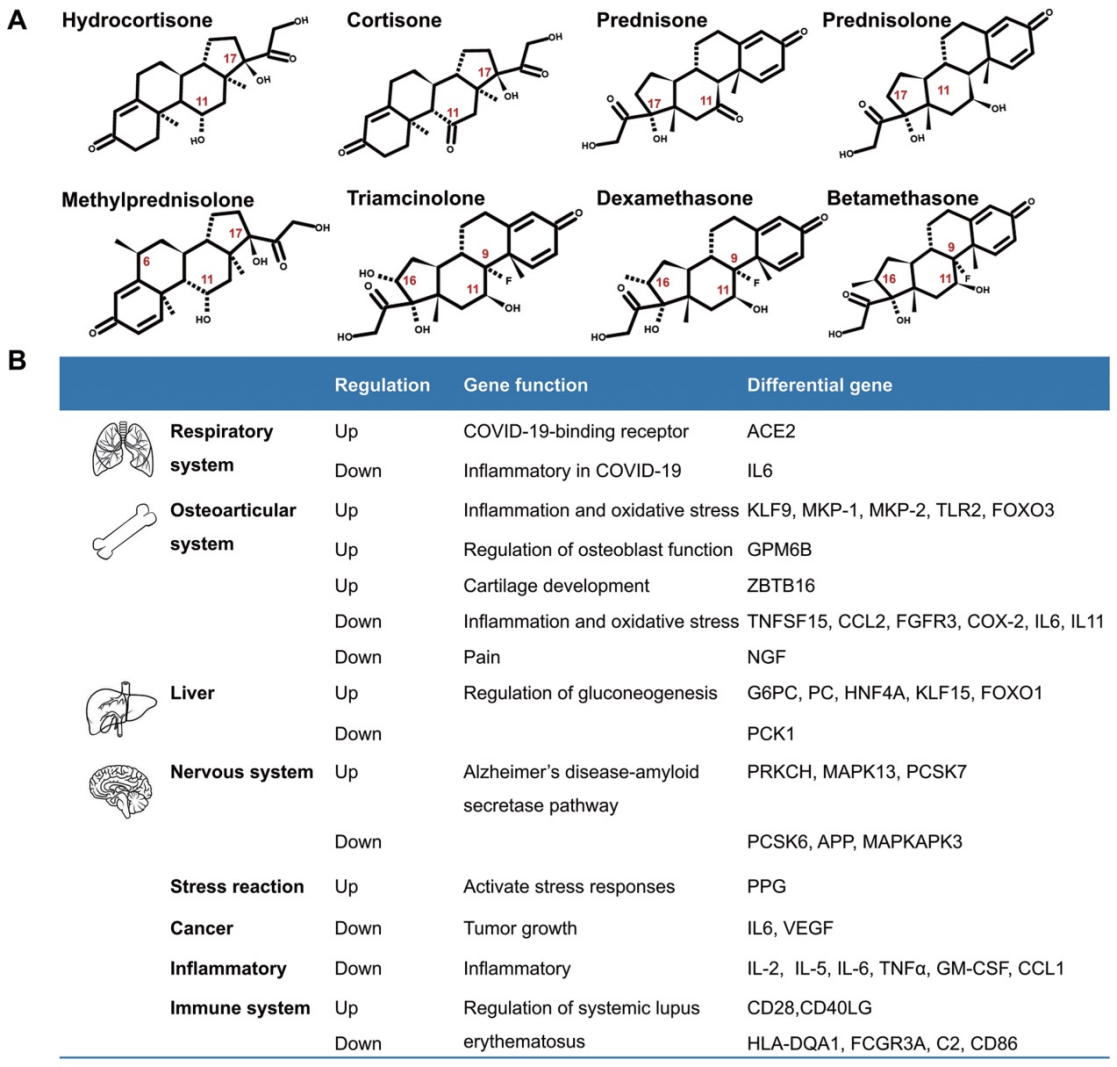
** Introduction of glucocorticoids.** A. The chemical structure of common glucocorticoids. B. The genes regulated by glucocorticoids in respiratory system, osteoarticular system, liver, nervous system, stress reaction, cancers, inflammation, Alzheimer disease and systemic lupus erythematosus, et al.

Although glucocorticoids have different names, they have similar biological effects. The commonly used glucocorticoids are prednisone, prednisolone, triamcinolone, methylprednisolone, dexamethasone, and others. They are modified in the chemical structures of the cortisone (hydrocortisone) backbone in order to enhance their anti-inflammatory effects. The chemical modifications include introduction of 6α-fluoro substitution, and reducing the binding to the mineralocorticoid receptor by insertion of a C=C double bond at C1, C2, or replacing a lipophilic substituent such as 21α-esters attached to the D-ring for increasing binding to glucocorticoid receptors 5 (Fig.1A). According to their duration of action, glucocorticoids are divided into short-acting, mid-acting, and long-acting drugs. The biological half-life of cortisone (hydrocortisone) is 8-12h, that of prednisone, prednisolone, methylprednisolone, and triamcinolone is 12-36h, and the biological half-life of dexamethasone and betamethasone is 36-54h (Table 1)^6^-^8^. The administration routes of glucocorticoids include external use, inhalation, oral and injection.

Glucocorticoids and GR have been an important area of research because of their pleiotropic physiological functions and extensive use in clinics. A number of genes are regulated by glucocorticoids (Fig. 1B). Recently, Pemmari and colleagues examined the global gene expression profiles of chondrocytes that were treated with dexamethasone for 24h by RNA-Seq. They found that dexamethasone affected lipid and glucose metabolism-related genes in addition to their anti-inflammatory, anticatabolic, and extracellular matrix-targeting effects 9. In human, many inflammatory genes are downstream genes of glucocorticoids. Escoter-Torres and Grbesa systematically summarized target genes based on glucocorticoid-GR binding. The broad physiological effects of glucocorticoid on the nervous (behavior, anxiety, visual), cardiovascular, immune, respiratory, reproductive, and musculoskeletal systems as well as on metabolism have been introduced 10. The well understanding of these genes will be helpful for proper usage of glucocorticoids 11.

## Double-edged sword concerns about glucocorticoids

Glucocorticoids are one of those medications that are regularly prescribed in both inpatient and outpatient departments. Their anti-inflammatory activity is generally used in treating various conditions, such as allergy, asthma, arthritis, inflammatory bowel disease, and cancer metastasis or exacerbation. However, they have numerous side effects, such as fluid retention, weight gain, and hyperglycemia, et al. With the long-term usage of glucocorticoids, especially long-term high-dose applications, a series of adverse events would appear. These adverse events include osteoporosis, hyperglycemia, insulin resistance, hypertension, muscle atrophy, severe infection, Cushing-like syndrome, peptic ulcers, and neuropsychiatric disorders. Actually, it is a real practical skill of a doctor to be able to properly use glucocorticoids. Cortisone (hydrocortisone) is physiologically similar to the internal hormone, and mainly used in the alternative treatment of adrenal insufficiency and other endocrine diseases. Considering the stronger anti-inflammatory effect and longer biological half-life, prednisolone, or methylprednisolone are mainly used in the treatment of rheumatoid immunologic diseases. Prednisone must be converted into prednisolone in liver, therefore, prednisolone should be chosen to avoid low treatment efficacy if patients have abnormal liver function. In critical conditions, such as sepsis, severe asthma, severe drug rashes, and acute nephritis, high doses of glucocorticoids are used intravenously. Glucocorticoids could not be used as simple antipyretic analgesic drugs. Glucocorticoids should be given locally when patients present with local inflammation, i.e., intra-joint injection is used for the treatment of arthritis, inhalation for asthma, and local application for dermatitis. The anti-inflammatory activities of classical glucocorticoids are useful for short-term use in most patients, but their chronic and systemic use usually causes side effects, and results in reducing sensitivity. The adverse effects of glucocorticoids are usually associated with high dose and long duration usage. Mazzantini and coworkers found that the treatment with 10 mg/d prednisone or equivalent for more than 3 months leads to a 7-fold increase in hip fractures and a 17-fold increase in vertebral fractures. Glucocorticoids are the most common cause of secondary osteoporosis, so-called glucocorticoid-induced osteoporosis 12. In addition, high dosage of glucocorticoids is immunosuppressive and can increase the risk of fatal infection. Therefore, giving optimal dosage of glucocorticoids at the right time is vital to control homeostasis, and avoid detrimental immunosuppression 13.

## Glucocorticoids and COVID-19 therapy

**Fig 2:**
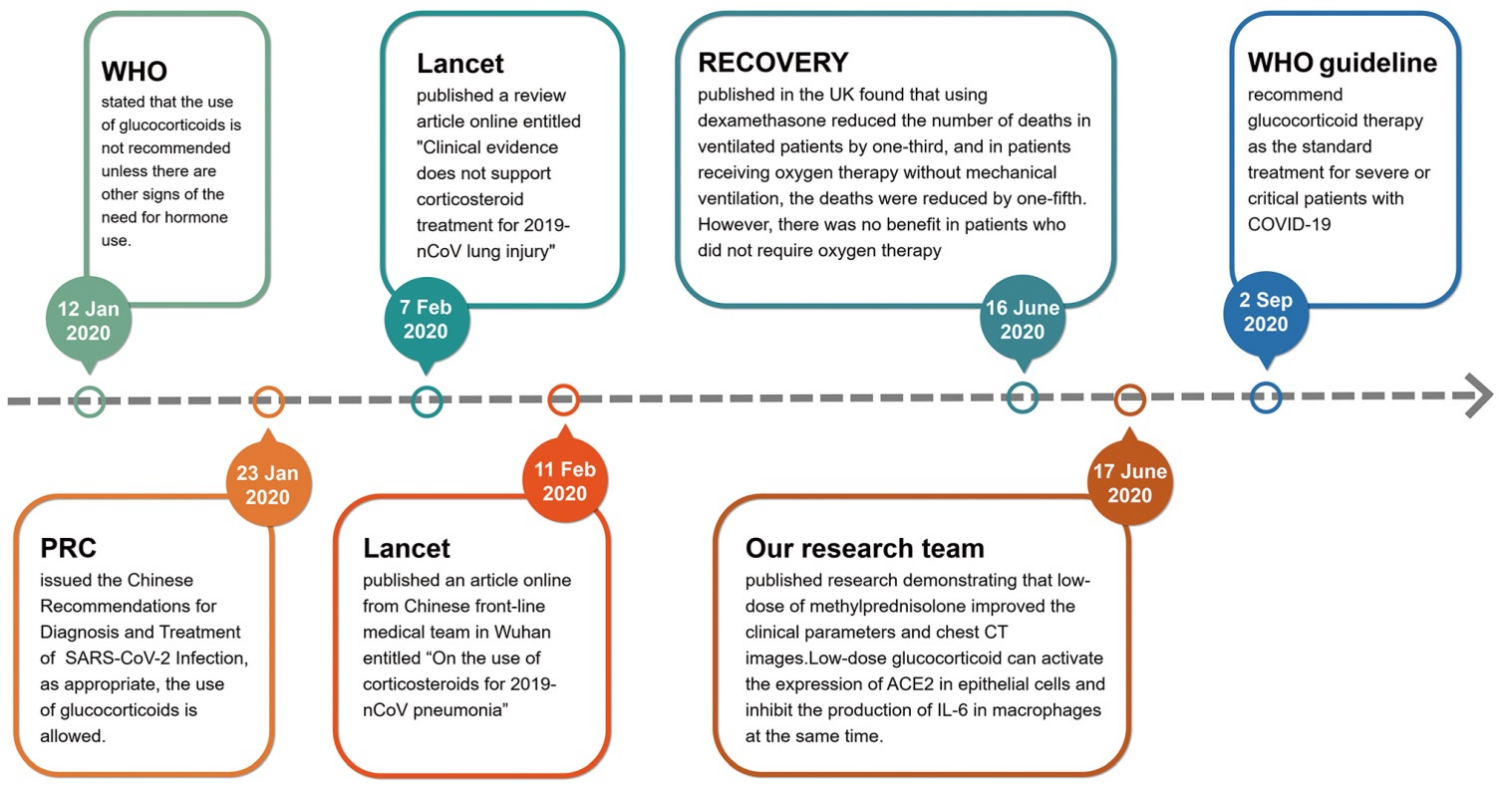
The flowchart of changed insights for glucocorticoids used in the treatment of severe COVID-19 since the pandemic.

In the early stages of the outbreak of COVID-19 in Wuhan, China, glucocorticoids were used cautiously. On 12 January 2020, the World Health Organization (WHO) issued the provisional guidelines for the treatment of severe acute respiratory infections suspected of 2019-nCoV, which stated that the use of glucocorticoids is not recommended unless there are other signs of the need for hormonal use (https://www.who.int/publications/i/item/10665-332299). On 7 February 2020, the Lancet published a review article online entitled "Clinical evidence does not support corticosteroid treatment for 2019-nCoV lung injury"^14^, but four days later, the Lancet published another article online from Chinese front-line medical team in Wuhan entitled “On the use of corticosteroids for 2019-nCoV pneumonia”^15^. Obviously, the use of glucocorticoids in the treatment of critical COVID-19 cases at the early stage of pandemic is controversial. According to the ongoing theory, glucocorticoids are the most important regulatory hormones in stress response. When a patient is faced with a serious stress like COVID-19, it is difficult for doctors to avoid using glucocorticoids 16. Therefore, doctors in various countries initiated a number of clinical trials to assess the efficacy of glucocorticoids 17-23. On 23 January 2020, the National Health Commission of PRC issued the Chinese Recommendations for Diagnosis and Treatment of Severe Novel Coronavirus (SARS-CoV-2) Infection (Trial version), which proposed that the use of glucocorticoids is allowed if appropriate (http://www.nhc.gov.cn/yzygj/s7653p/202001/9fbefc9a5fe747e98ea5baeedfb68158.shtml). The results of clinical trials on the benefits of using glucocorticoids in the treatment of patients with COVID-19 were gradually published in mid-2020. The most famous clinical trial result of Randomised Evaluation of COVID-19 Therapy (RECOVERY) was published in the N Engl J Med by doctors of the United Kingdom 24, 25. They found that using dexamethasone (6 mg/day, once daily for up to 10 days) reduced the number of deaths in ventilated patients by one-third, and in patients receiving oxygen therapy without mechanical ventilation, the deaths were reduced by one-fifth in adults patients with COVID-19. However, there was no benefit (and the possibility of harm) in patients who did not require oxygen therapy 24. Simultaneously, our research team published a research result entitled “Glucocorticoids improve severe or critical COVID-19 through activating ACE2 and reducing IL-6 levels” 26. This research demonstrated that low-dose of methylprednisolone (40 mg/d if body weight ≤ 80 kg for the first 3-4 days, and then 20 mg/d for the next 3 days or more with a total of less than 8 days) improved the clinical tests and chest CT images in 7 out of 9 patients of severe or critical COVID-19. We focused on the molecular target angiotensin-converting enzyme 2 (ACE2) of SARS-CoV-2 and screened a group of ACE2 agonists by bioinformatics. Glucocorticoids are a type of ACE2 activator. We further verified the efficacy of nine chemicals on regulating ACE2 expression in human GES-1, an upper digestive tract epithelial cell line, and THP-1, a human monocyte cell line, and found that several glucocorticoids possessed activating effects on ACE2 in both cell lines. Our group also compared the efficacies of several glucocorticoids. Hydrocortisone showed the strongest effect on ACE2 activation, followed by prednisolone, and methylprednisolone 26. Given their powerful anti-inflammatory and immunomodulatory effects, glucocorticoids are an obvious potential therapy for acute lung injury in severe COVID-19^27^. Along with the efficacy in treating the acute lung injury / acute respiratory distress syndrome (ARDS), glucocorticoids have been proposed as a life-saving drug for treating COVID-19^25^, ^28^. On 2 September 2020, the standard treatment for severe or critical patients with COVID-19 was recommended by the WHO guideline (https://www.who.int/publications/i/item/WHO-2019-nCoV-Corticosteroids-2020.1) (Fig. 2). However, the limitation of RECOVERY study is that the side effects of glucocorticoids are not adequately assessed.

Thereafter, the efficacy of glucocorticoids in the treatment of COVID-19 has been reported by different groups from different countries 29-31. Keller et al. reported that glucocorticoids could significantly reduce the risk of mortality or mechanical ventilation (odds ratio, 0.23; 95% CI, 0.08-0.70) if the initial level of C-reactive protein (CRP) is >/=20 mg/dl. However, using glucocorticoids would increase the risk of mortality or mechanical ventilation (OR, 2.64; 95% CI, 1.39-5.03) if the CRP level is less than 10 mg/dl 32. On 5 January, 2021, Chinese researchers proposed the use of glucocorticoids for COVID-19 treatment. They used the ratio of neutrophils to lymphocytes (NLR) as an evaluation index to determine the clinical efficacy of glucocorticoids. They analyzed 12,862 COVID-19 cases from 21 hospitals in Hubei Province, and found that NLR > 6.11 at admission is a risk factor for mortality. However, glucocorticoids treatment in such individuals is associated with a low 60-day all-cause mortality. Conversely, in individuals with an NLR </= 6.11 or with type 2 diabetes, glucocorticoids treatment was not associated with reduced mortality, but rather increased risks of hyperglycemia and infections^33^. Recently, Liu and coworkers proposed that administration of glucocorticoids in severe COVID-19 is associated with increased 28-day mortality and delayed SARS-CoV-2 coronavirus RNA clearance 34. However, Ji et al. reported that glucocorticoid therapy does not delay viral clearance in COVID-19 patients 35. To analyze the effect of glucocorticoids on patients with severe pneumonia, Liu and colleagues found that there was no significant difference between patients using low dose (</= 2 mg/kg day) and high dose (> 2 mg/kg day) methylprednisolone in inhibiting IL-6 production. There was no significant difference in virus clearance between patients with and without methylprednisolone use 36. Ai and coworkers proposed that glucocorticoids could be used in severe or critical illness. According to the experience of treating COVID-19 patients in Shanghai, the dose of methylprednisolone was 40-80 mg/day for 3 days, and then reduced to 20 mg/day, with a total treatment period of less than 7 days. The side effects are minimum with this dose regimen 37.

## Glucocorticoids and treatment of solid tumors

Glucocorticoids have been used in clinical oncology for over half a century for alleviating side effects induced by chemotherapy or radiotherapy. As a supportive therapy, glucocorticoids are frequently used for increasing appetite, reducing weight loss, eliminating fatigue, and preventing vomiting 38. Glucocorticoids are also effective in alleviating pain associated with bone metastasis by inhibiting the synthesis and release of prostaglandins in advanced cancer patients 39. However, a meta-analysis disclosed that the use of glucocorticoids should be avoided in patients with advanced cancers, because it may reduce survival of patients with lung cancer as palliative/supportive care purpose 40.

Currently, immune checkpoint inhibitor (ICPI) therapy used in cancer treatment exerts its effect by blocking the actions of cytotoxic T-lymphocyte-associated protein 4 (CTLA-4) and programmed cell death protein 1 (PD-1). ICPIs can enhance antitumor immunity by blocking negative regulators of T-cell function that exist on both immune cells and tumor cells. Although these agents can lead to remarkable responses, they are also associated with immune-related adverse effects (irAEs). Friedman and colleagues proposed that PD-1 inhibitors such as nivolumab and pembrolizumab have a lower incidence of irAEs compared with those that block CTLA-4 such as ipilimumab. The combination of nivolumab and ipilimumab has a higher rate of irAEs than either approach as monotherapy. The most common irAEs include rash, colitis, hepatitis, endocrinopathies, and pneumonitis. The treatment of irAEs requires immunosuppression with glucocorticoids 41, because ICPI results in an increase of effector T cells, that respond to self-antigen, and lead to autoimmunity. The degree of autoimmune effect may be correlated with tumor response to immunotherapy. ICPI could enhance the anti-tumor effects of T-cells, but also enhance the immune response to normal tissues 42. On October 2015, Horvat and colleagues reported their clinical findings in patients with melanoma who had received ipilimumab treatment at the standard dose of 3 mg/kg, and observed irAEs. Of the 298 patients, 254 (85%) experienced irAEs of any grade, and 56 (19%) discontinued therapy because of irAEs, most commonly diarrhea. A total of 103 (35%) patients received treatment with systemic glucocorticoids. They suggested that irAEs are common in patients treated with ipilimumab. Based on their experience, approximately one-third of ipilimumab-treated patients required treatment with systemic glucocorticoids 43. Arbour and coworkers reported that there were 90(14%) of 640 patients of non-small-cell lung cancer treated with single-agent PD-(L) 1 blockade who also received glucocorticoids of >/= 10 mg of prednisone equivalent daily at the start of the PD-(L)1 blockade therapy. The common indications for corticosteroids were dyspnea (33%), fatigue (21%), and brain metastases. They suggested that the baseline use of glucocorticoids >/= 10 mg of prednisone or equivalent was associated with poor outcome in patients with non-small-cell lung cancer who were treated with PD-(L)1 blockade. Prudent use of glucocorticoids at the time of initiating PD-(L)1 blockade is recommended 44.

The two-sided sword concerns of using glucocorticoids also existed in cancer treatment. Taking the widely used dexamethasone as an example, this drug belongs to the long-acting glucocorticoid, which has stronger anti-inflammatory, anti-allergic and anti-toxic effects than prednisone (mid-acting glucocorticoid). Thus, dexamethasone is only suitable for short-term usage. The long-term application of dexamethasone can lead to iatrogenic adrenal insufficiency 45. In addition, if malignant tumor cells overexpressed glucocorticoid receptor, using glucocorticoids should be careful, because overexpression of glucocorticoid receptor may promote the proliferation of malignant cancer cells 46, 47. Theoretically, the effects of glucocorticoids are mediated by the ubiquitously expressed glucocorticoid receptor, an intracellular ligand-activated transcription factor. Upon glucocorticoid binding, the glucocorticoid receptor becomes hyperphosphorylated. The hyperphosphorylated receptor subsequently migrates to the nucleus, where the glucocorticoid receptor can bind to DNA directly to regulate transcription of target genes 48. Tian and colleagues analyzed the effects of expression of glucocorticoid receptor on colorectal carcinoma cell lines HT29 (primary cell line) and T84 (metastatic cell line). They found that the expression of glucocorticoid receptor were significantly upregulated in nucleus of metastatic T84 cells. Dexamethasone treatment caused increased proliferation and invasion in T84 cell, compared to HT29 cell through activating CDK1 expression. Depletion of glucocorticoid receptor suppressed proliferation of metastatic colon carcinoma cells. Depletion of CDK1 also suppressed the proliferation and invasion of T84 cells. This study suggests that glucocorticoid- glucocorticoid receptor -CDK1 pathway induces proliferation and invasion of colon cancer cells 49.

On the other hand, some studies found that glucocorticoids could suppress cancer proliferation through activating glucocorticoid receptor 50, 51. For example, Yao and coworkers recently demonstrated that dexamethasone inhibits pancreatic tumor growth in preclinical models. They examined the expression of glucocorticoid receptor of two pancreatic cancer cell lines, PANC-1 and SW1990, and found that abundant expression of glucocorticoid receptor in PANC-1cells. Dexamethasone treatment significantly inhibited colony formation, migration, and tumor growth of PANC-1 cells. The underlying mechanisms involved in suppression of phosphorylation of nuclear factor κB and downregulation of epithelial-to-mesenchymal transition, interleukin 6 and vascular endothelial growth factor. Their findings suggest that dexamethasone effectively inhibits pancreatic tumor growth partially through activation of glucocorticoid receptor. The potential correlation between glucocorticoid receptor expression and anti-cancer efficacy of dexamethasone provided a new direction for developing target therapy 52.

In conclusions, glucocorticoids are important hormones in response to internal or environmental stresses. However, chronic glucocorticoid use can result in detrimental side effects, including osteoporosis, hyperglycemia, diabetes mellitus, obesity, cardiovascular diseases and depression. Therefore, attention should be given on the dual roles of glucocorticoids. In patients with severe or critical COVID-19, glucocorticoids are ideally administered for short-term use for their classical anti-inflammatory activities. Chronic and systemic use of glucocorticoids should be avoided, because they can profoundly affect bone cell function and mineral metabolism, and lead to osteoporosis 53. Clinically, glucocorticoids may improve some symptoms, quality of life of patients with advanced cancer. However, the long-term glucocorticoid therapy in cancers is associated with adverse effects.

## Figures and Tables

**Table 1:** Comparison of anti-inflammatory function, equivalent doses, plasma half-life and acting duration of several glucocorticoids

Category	Drug	Effect of anti-inflammation (ratio)*	Equivalent dose(mg)*	Plasma half-life (min.)	Duration of action(h)
Short-acting	Hydrocortisone	1	20	90	8-12
	Cortisone	0.8	25	30	8-12
Mid-acting	Prednisone	3.5	5	60	12-36
	Prednisolone	4.0	5	200	12-36
	Methylprednisolone	5.0	4	180	12-36
	Triamcinolone	5.0	4	>200	12-36
Long-acting	Dexamethasone	30	0.75	100-300	36-54
	Betamethasone	25-35	0.6	100-300	36-54

* The ratio of anti-inflammatory effects is measured by the reference of hydrogenation as 1. The equivalent dose is based on the hydrogenated as reference.

## References

[1] Vandewalle J, Luypaert A, De Bosscher K, Libert C (2018). Therapeutic Mechanisms of Glucocorticoids. Trends Endocrinol Metab.

[2] Timmermans S, Souffriau J, Libert C (2019). A General Introduction to Glucocorticoid Biology. Front Immunol.

[3] Alessi J, de Oliveira GB, Schaan BD, Telo GH (2020). Dexamethasone in the era of COVID-19: friend or foe? An essay on the effects of dexamethasone and the potential risks of its inadvertent use in patients with diabetes. Diabetol Metab Syndr.

[4] Barnes PJ (2011). Glucocorticosteroids: current and future directions. Br J Pharmacol.

[5] Adcock IM, Mumby S (2017). Glucocorticoids. Handb Exp Pharmacol.

[6] Czock D, Keller F, Rasche FM, Haussler U (2005). Pharmacokinetics and pharmacodynamics of systemically administered glucocorticoids. Clin Pharmacokinet.

[7] Daley-Yates PT (2015). Inhaled corticosteroids: potency, dose equivalence and therapeutic index. Br J Clin Pharmacol.

[8] Bledsoe RK, Montana VG, Stanley TB, Delves CJ, Apolito CJ, McKee DD (2002). Crystal structure of the glucocorticoid receptor ligand binding domain reveals a novel mode of receptor dimerization and coactivator recognition. Cell.

[9] Pemmari A, Leppanen T, Hamalainen M, Moilanen T, Vuolteenaho K, Moilanen E (2020). Widespread regulation of gene expression by glucocorticoids in chondrocytes from patients with osteoarthritis as determined by RNA-Seq. Arthritis Res Ther.

[10] Grbesa I, Hakim O (2017). Genomic effects of glucocorticoids. Protoplasma.

[11] Escoter-Torres L, Caratti G, Mechtidou A, Tuckermann J, Uhlenhaut NH, Vettorazzi S (2019). Fighting the Fire: Mechanisms of Inflammatory Gene Regulation by the Glucocorticoid Receptor. Front Immunol.

[12] Mazzantini M, Di Munno O (2014). Glucocorticoid-induced osteoporosis: 2013 update. Reumatismo.

[13] Vedamurthy A, Xu L, Luther J, Colizzo F, Garber JJ, Khalili H (2018). Long-Term Outcomes of Immunosuppression-Naive Steroid Responders Following Hospitalization for Ulcerative Colitis. Dig Dis Sci.

[14] Russell CD, Millar JE, Baillie JK (2020). Clinical evidence does not support corticosteroid treatment for 2019-nCoV lung injury. Lancet.

[15] Shang L, Zhao J, Hu Y, Du R, Cao B (2020). On the use of corticosteroids for 2019-nCoV pneumonia. Lancet.

[16] Castelli V, Cimini A, Ferri C (2020). Cytokine Storm in COVID-19: "When You Come Out of the Storm, You Won't Be the Same Person Who Walked in". Front Immunol.

[17] Maskin LP, Olarte GL, Palizas F Jr, Velo AE, Lurbet MF, Bonelli I (2020). High dose dexamethasone treatment for Acute Respiratory Distress Syndrome secondary to COVID-19: a structured summary of a study protocol for a randomised controlled trial. Trials.

[18] Zhu HM, Li Y, Li BY, Yang S, Peng D, Yang X (2020). Effect of methylprednisolone in severe and critical COVID-19: Analysis of 102 cases. World J Clin Cases.

[19] Borie R, Savale L, Dossier A, Ghosn J, Taille C, Visseaux B (2020). Glucocorticoids with low-dose anti-IL1 anakinra rescue in severe non-ICU COVID-19 infection: A cohort study. PLoS One.

[20] Callejas Rubio JL, Luna Del Castillo JD, de la Hera Fernandez J, Guirao Arrabal E, Colmenero Ruiz M, Ortego Centeno N (2020). Effectiveness of corticoid pulses in patients with cytokine storm syndrome induced by SARS-CoV-2 infection. Med Clin (Engl Ed).

[21] Sterne JAC, Diaz J, Villar J, Murthy S, Slutsky AS, Perner A (2020). Corticosteroid therapy for critically ill patients with COVID-19: A structured summary of a study protocol for a prospective meta-analysis of randomized trials. Trials.

[22] Qin YY, Zhou YH, Lu YQ, Sun F, Yang S, Harypursat V (2020). Effectiveness of glucocorticoid therapy in patients with severe coronavirus disease 2019: protocol of a randomized controlled trial. Chin Med J (Engl).

[23] Hu Y, Wang T, Hu Z, Wang X, Zhang Z, Li L (2020). Clinical efficacy of glucocorticoid on the treatment of patients with COVID-19 pneumonia: A single-center experience. Biomed Pharmacother.

[24] Group RC, Horby P, Lim WS, Emberson JR, Mafham M, Bell JL (2020). Dexamethasone in Hospitalized Patients with Covid-19 - Preliminary Report. N Engl J Med.

[25] Ledford H (2020). Coronavirus breakthrough: dexamethasone is first drug shown to save lives. Nature.

[26] Xiang Z, Liu J, Shi D, Chen W, Li J, Yan R (2020). Glucocorticoids improve severe or critical COVID-19 by activating ACE2 and reducing IL-6 levels. Int J Biol Sci.

[27] Moore JB, June CH (2020). Cytokine release syndrome in severe COVID-19. Science.

[28] Deng CX (2020). Glucocorticoids save lives in COVID-19 patients. Int J Biol Sci.

[29] Fernandez-Cruz A, Ruiz-Antoran B, Munoz-Gomez A, Sancho-Lopez A, Mills-Sanchez P, Centeno-Soto GA (2020). A Retrospective Controlled Cohort Study of the Impact of Glucocorticoid Treatment in SARS-CoV-2 Infection Mortality. Antimicrob Agents Chemother.

[30] Han D, Peng C, Meng R, Yao J, Zhou Q, Xiao Y (2020). Estimating the release of inflammatory factors and use of glucocorticoid therapy for COVID-19 patients with comorbidities. Aging (Albany NY).

[31] Fu HY, Luo Y, Gao JP, Wang L, Li HJ, Li X (2020). Effects of Short-Term Low-Dose Glucocorticoids for Patients with Mild COVID-19. Biomed Res Int.

[32] Keller MJ, Kitsis EA, Arora S, Chen JT, Agarwal S, Ross MJ (2020). Effect of Systemic Glucocorticoids on Mortality or Mechanical Ventilation in Patients With COVID-19. J Hosp Med.

[33] Cai J, Li H, Zhang C, Chen Z, Liu H, Lei F (2021). The Neutrophil-to-Lymphocyte Ratio Determines Clinical Efficacy of Corticosteroid Therapy in Patients with COVID-19. Cell Metab.

[34] Liu J, Zhang S, Dong X, Li Z, Xu Q, Feng H (2020). Corticosteroid treatment in severe COVID-19 patients with acute respiratory distress syndrome. J Clin Invest.

[35] Ji J, Zhang J, Shao Z, Xie Q, Zhong L, Liu Z (2020). Glucocorticoid therapy does not delay viral clearance in COVID-19 patients. Crit Care.

[36] Liu F, Ji C, Luo J, Wu W, Zhang J, Zhong Z (2020). Clinical characteristics and corticosteroids application of different clinical types in patients with corona virus disease 2019. Sci Rep.

[37] Ai J, Li Y, Zhou X, Zhang W (2020). COVID-19: treating and managing severe cases. Cell Res.

[38] Morrow GR, Shelke AR, Roscoe JA, Hickok JT, Mustian K (2005). Management of cancer-related fatigue. Cancer Invest.

[39] Lin KT, Wang LH (2016). New dimension of glucocorticoids in cancer treatment. Steroids.

[40] Petrelli F, Bukovec R, Perego G, Luisa R, Luciani A, Zaniboni A (2020). Association of steroid use with survival in solid tumours. Eur J Cancer.

[41] Friedman CF, Proverbs-Singh TA, Postow MA (2016). Treatment of the Immune-Related Adverse Effects of Immune Checkpoint Inhibitors: A Review. JAMA Oncol.

[42] Marinelli D, Giusti R, Mazzotta M, Filetti M, Krasniqi E, Pizzuti L (2021). Palliative- and non-palliative indications for glucocorticoids use in course of immune-checkpoint inhibition. Current evidence and future perspectives. Crit Rev Oncol Hematol.

[43] Horvat TZ, Adel NG, Dang TO, Momtaz P, Postow MA, Callahan MK (2015). Immune-Related Adverse Events, Need for Systemic Immunosuppression, and Effects on Survival and Time to Treatment Failure in Patients With Melanoma Treated With Ipilimumab at Memorial Sloan Kettering Cancer Center. J Clin Oncol.

[44] Arbour KC, Mezquita L, Long N, Rizvi H, Auclin E, Ni A (2018). Impact of Baseline Steroids on Efficacy of Programmed Cell Death-1 and Programmed Death-Ligand 1 Blockade in Patients With Non-Small-Cell Lung Cancer. J Clin Oncol.

[45] Skov M, Main KM, Sillesen IB, Muller J, Koch C, Lanng S (2002). Iatrogenic adrenal insufficiency as a side-effect of combined treatment of itraconazole and budesonide. Eur Respir J.

[46] Zhidkova EM, Lylova ES, Savinkova AV, Mertsalov SA, Kirsanov KI, Belitsky GA (2020). A Brief Overview of the Paradoxical Role of Glucocorticoids in Breast Cancer. Breast Cancer (Auckl).

[47] Ayroldi E, Cannarile L, Delfino DV, Riccardi C (2018). A dual role for glucocorticoid-induced leucine zipper in glucocorticoid function: tumor growth promotion or suppression?. Cell Death Dis.

[48] Flaherty RL, Intabli H, Falcinelli M, Bucca G, Hesketh A, Patel BA (2019). Stress hormone-mediated acceleration of breast cancer metastasis is halted by inhibition of nitric oxide synthase. Cancer Lett.

[49] Tian D, Tian M, Han G, Li JL (2019). Increased glucocorticoid receptor activity and proliferation in metastatic colon cancer. Sci Rep.

[50] Dobos J, Kenessey I, Timar J, Ladanyi A (2011). Glucocorticoid receptor expression and antiproliferative effect of dexamethasone on human melanoma cells. Pathol Oncol Res.

[51] Kostopoulou ON, Mohammad AA, Bartek J Jr, Winter J, Jung M, Stragliotto G (2018). Glucocorticoids promote a glioma stem cell-like phenotype and resistance to chemotherapy in human glioblastoma primary cells: Biological and prognostic significance. Int J Cancer.

[52] Yao Y, Yao QY, Xue JS, Tian XY, An QM, Cui LX (2020). Dexamethasone inhibits pancreatic tumor growth in preclinical models: Involvement of activating glucocorticoid receptor. Toxicol Appl Pharmacol.

[53] Tang J (2020). What can we learn about corticosteroid therapy as a treatment for COVID-19?. Osteoporos Int.

